# Premature Constriction of Fetal Ductus Arteriosus Caused by Sertraline in a Pregnant Woman: A Case Report

**DOI:** 10.1002/ccr3.70074

**Published:** 2025-01-06

**Authors:** Alireza Golbabaei, Elham sadat Alavi moghaddam, Hooman Mohammad Talebi

**Affiliations:** ^1^ Pediatric Cardiologist, Department of Perinatology and Fetal Cardiology Shariati Hospital, Tehran University of Medical Sciences Tehran Iran; ^2^ Obstetric and Gynecologist Tehran University of Medical Sciences Tehran Iran; ^3^ Faculty Member at Nursing Department Khomein University of Medical Sciences Khomein Iran

**Keywords:** case report, ductus arteriosus, pregnancy, pregnant woman, sertraline

## Abstract

Fetal ductus arteriosus was treated in a 39‐year‐old pregnant woman in the 33rd week After psychiatric consultation and discontinuation of sertraline which underscores the association of sertraline with premature ductus arteriosus constriction.

## 1 Introduction

Premature constriction of the patent ductus arteriosus (PDA) is a rare condition that can occur during pregnancy. The ductus arteriosus is a blood vessel that connects the pulmonary artery to the aorta, allowing blood to bypass the lungs during fetal development. When this vessel closes prematurely, it can lead to decreased blood flow to the lungs and other organs, potentially resulting in fetal heart failure or death [1, 2]. The studies show that the incidence of the patent ductus arteriosus is 1 in 2000 in term neonates, which indicates the rarity of the condition [3]. On the other hand, the incidence of this condition is reported as 10%–14.4% among all congenital abnormalities [4].

The association between Selective Serotonin Reuptake Inhibitors (SSRIs) and the Risk of Congenital Heart Defects is discussed in recent studies [5]. Sertraline, as a selective serotonin reuptake inhibitor (SSRI) commonly used to treat depression and anxiety, has been shown to have a vasoconstriction effect on the ductus arteriosus in animal studies [6]. Additionally, human studies indicate that SSRIs prescription during pregnancy can cause cardiovascular disorders and admission in intensive care units [7]. Accordingly, the prescription of Selective Serotonin Re‐Uptake Inhibitors (SSRIs) is reported as a potential cause of fetal ductal constriction [8].

In the present study, we report a case of premature constriction of fetal ductus arteriosus in a pregnant woman who was treated after psychiatric consultation and discontinuing the sertraline by the psychiatrist at the 33rd week of gestational age.

## 2 Case History/Examination

A 39 years old, 2 gravid, 1 abortion, and 0 living child pregnant woman at the 33rd week of gestational age was referred to our hospital with a report of sonography in which fetal cardiomegaly was reported. The patient gave a history of Depression disorder during the last 8 years and was being treated with sertraline according to the physician's prescription. There were no other maternal risk factors or history of other diseases. Also, the patient did not report any specific drug history or family history. Routine laboratory tests were normal and the physician did not request any specific serum tests.

## 3 Methods

Fetal echocardiography was immediately performed for the patient, and the initial diagnosis was confirmed. Finally, and based on all findings of fetal ultrasound, premature fetal ductus arteriosus constriction was documented. The pregnancy continued and a complete history was taken from the patient in terms of consumption of any specific drugs and foods. Finally, the chronic usage of sertraline, 100 mg daily, was considered the most likely factor of involvement of the fetal cardiovascular system.

Psychiatric consultation was requested, and according to the order of the psychiatrist, this drug was discontinued and another drug from another category was replaced. The patient followed every 2–7 days and fetal echocardiography was performed for the patient on every visit. Ultrasound findings showed a gradual decline in velocity (stenosis) of the ductus arteriosus‐descending aorta connection location. After about 3 weeks, abnormal echocardiographic findings completely reversed to the normal state. Pregnancy was terminated at 39 weeks of gestational age and with normal vaginal delivery. After birth, the general condition of the patient was good and after some hours the baby was delivered to the mother. For further follow‐ups, the neonate was assessed once a week for 2 months after birth to ensure complete closure of the duct and persistent pulmonary hypertension. At the end of the second month after birth, an echocardiography was performed and no abnormality was seen.

**FIGURE 1:**
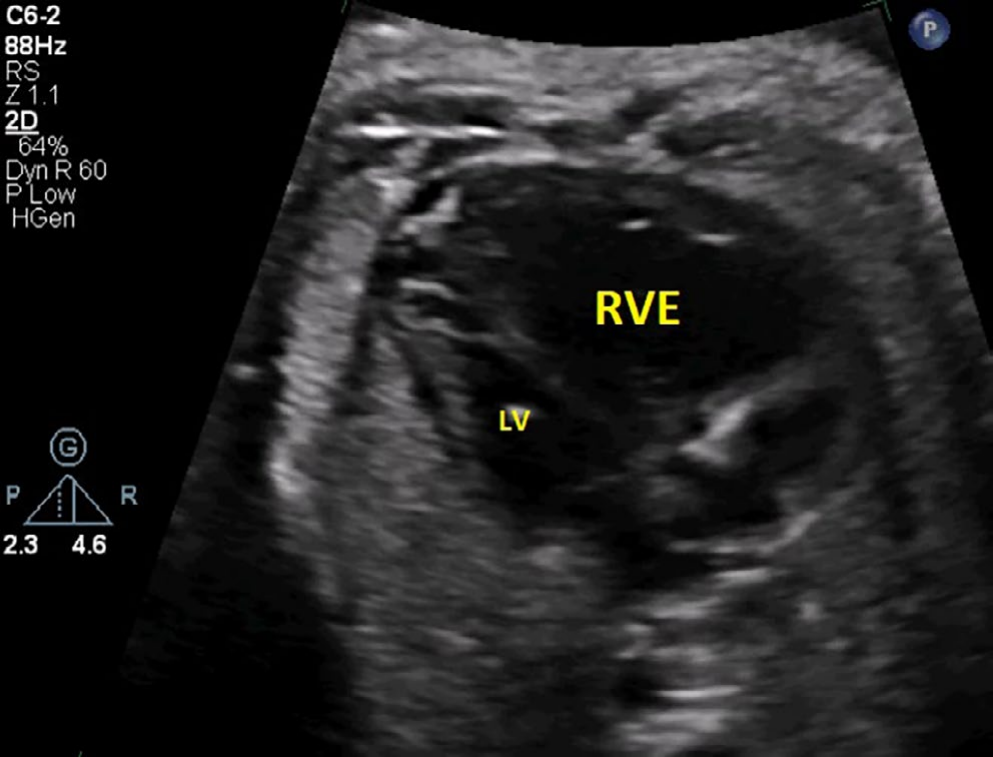
The four‐chamber view of the fetal heart demonstrates right ventricular enlargement (RVE) with a notable size discrepancy between the right ventricle (RV) and left ventricle (LV).

**FIGURE 2:**
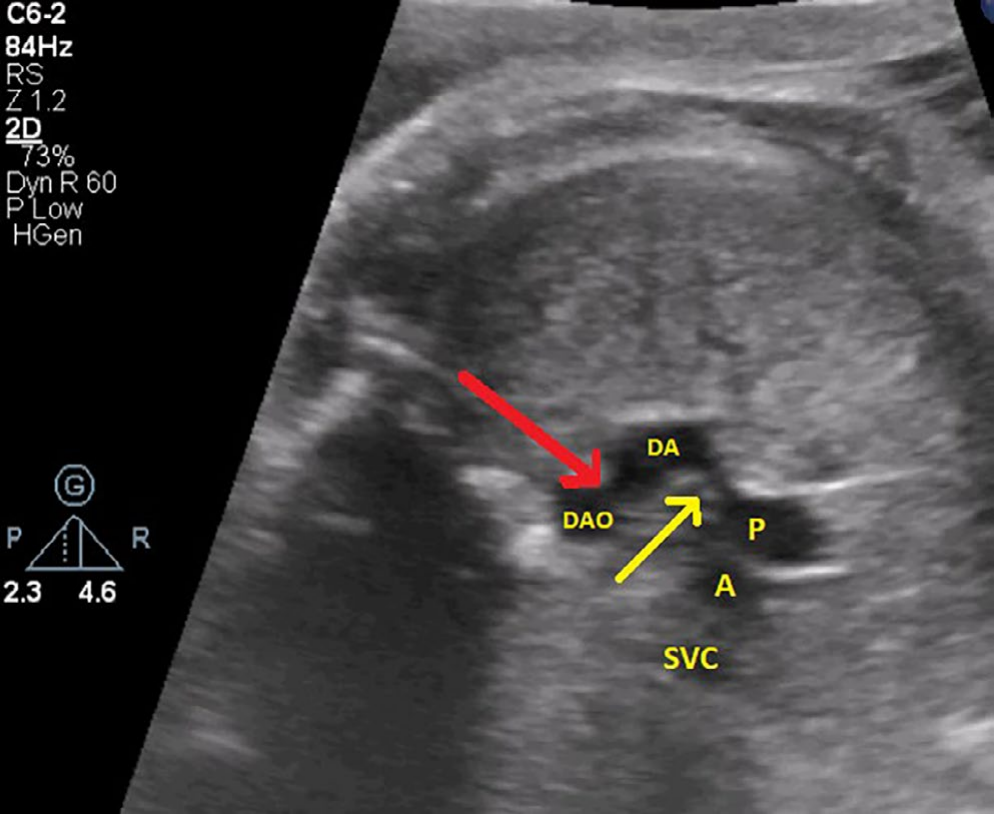
Axial imaging of the cardiac vessels reveals an S‐shaped ductus arteriosus. Notable stenosis is observed at two critical sites: the connection between the pulmonary artery (P) and the ductus arteriosus (DA) (indicated by the yellow arrow) and the junction of the ductus arteriosus with the descending aorta (DAO) (indicated by the red arrow).

**FIGURE 3:**
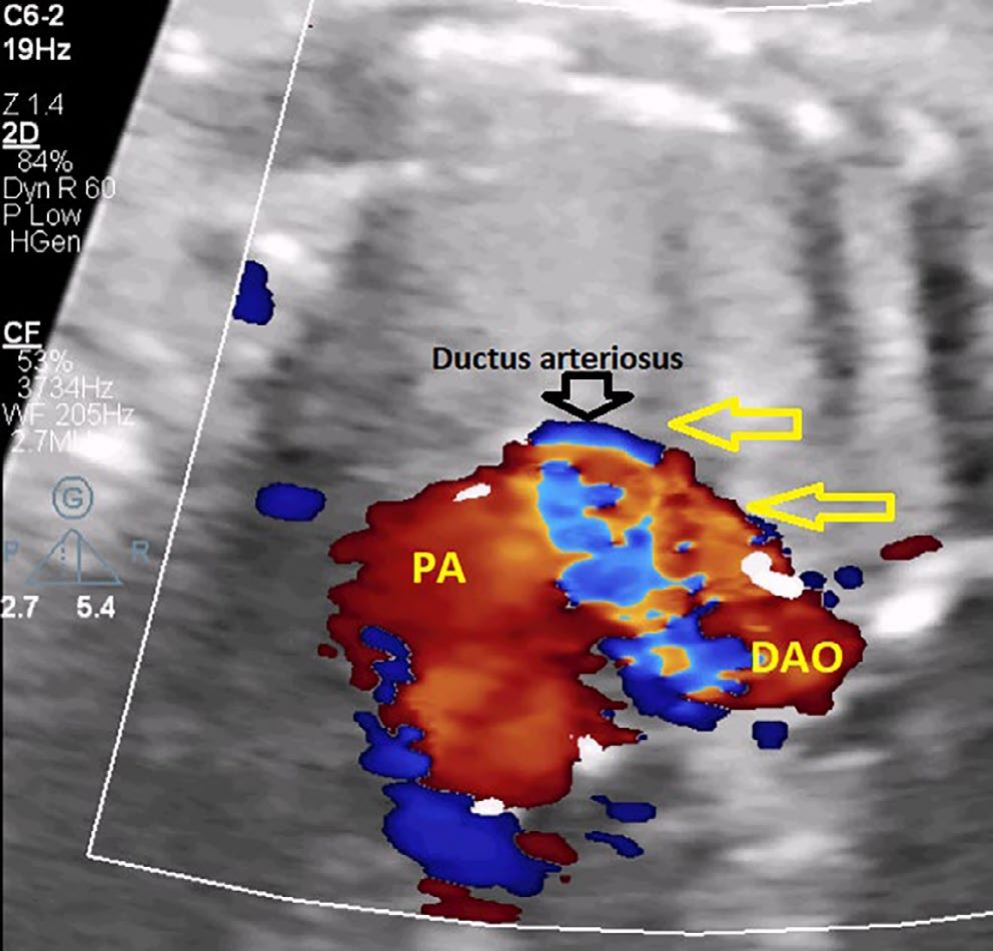
The four‐chamber view with color Doppler imaging demonstrates flow acceleration, evidenced by aliasing.

**FIGURE 4:**
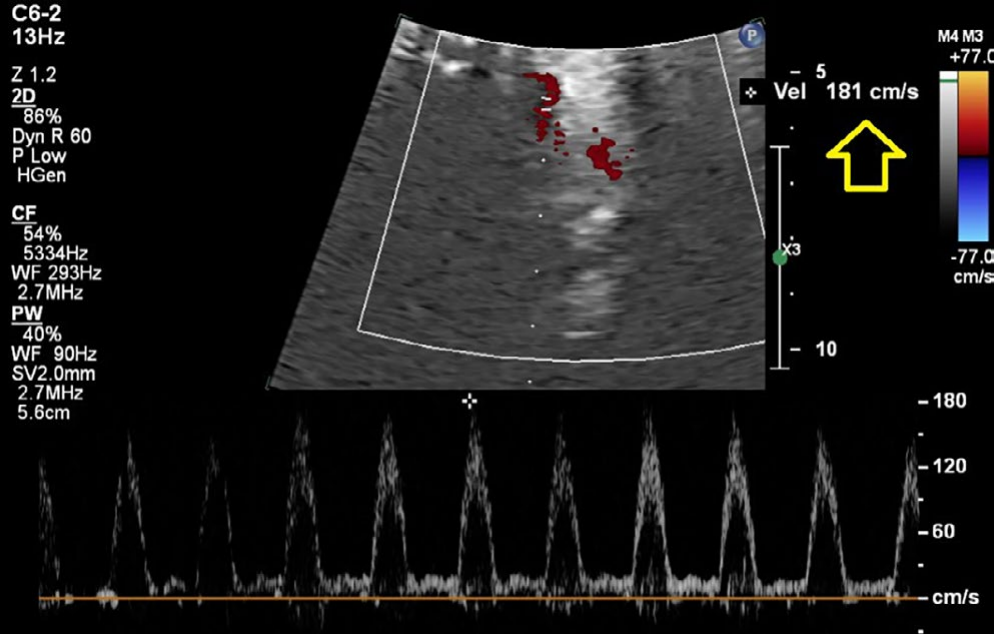
Spectral Doppler interrogation at the stenotic segment of the ductus arteriosus reveals elevated peak flow velocity, consistent with hemodynamic narrowing.

According to the provided figures, four‐chamber view of the fetal heart shows right ventricular enlargement and a smaller left ventricle (Figure 1). Axial view of the great vessels shows a typical S‐shaped ductus arteriosus (due to constriction) and stenotic ductus arteriosus (Figure 2). Significant flow acceleration (aliasing) due to ductus arteriosus stenosis has been shown in Figure 3. In Figure 4, high velocity (significant stenosis) in DA‐DAO connection point is clear.

## 4 Conclusion and Results

**FIGURE 5:**
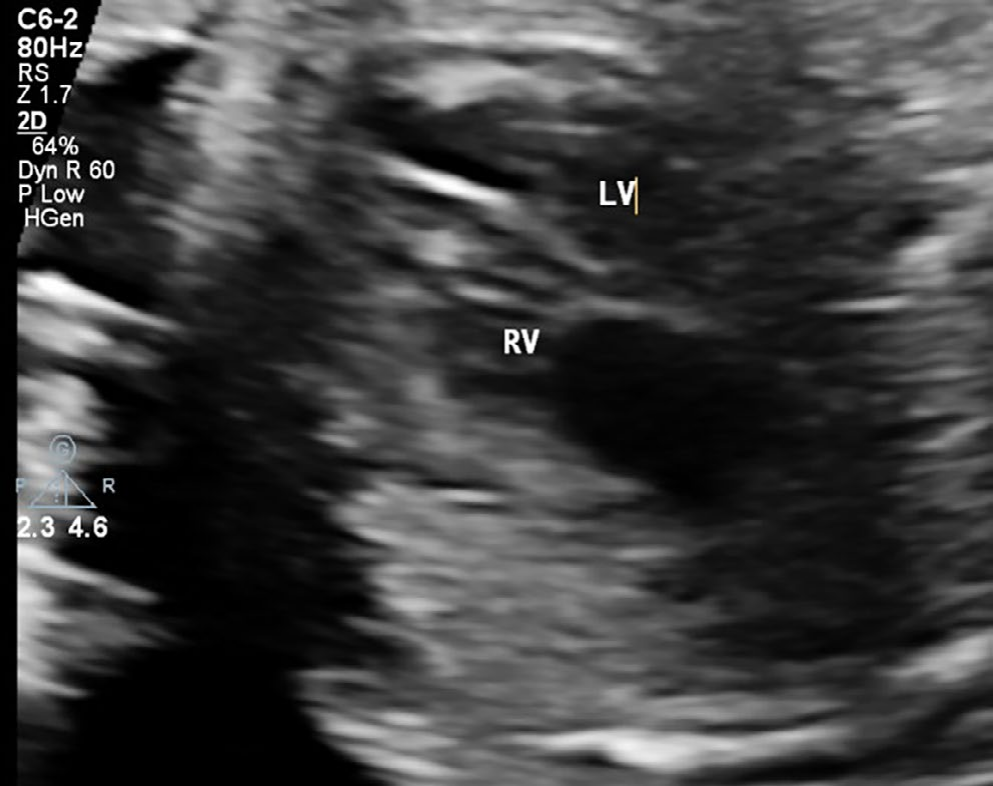
Axial fetal ultrasound demonstrates normalization of right ventricular size and resolution of the previously noted size discrepancy between the right ventricle (RV) and left ventricle (LV), with reversal of the disproportion.

**FIGURE 6:**
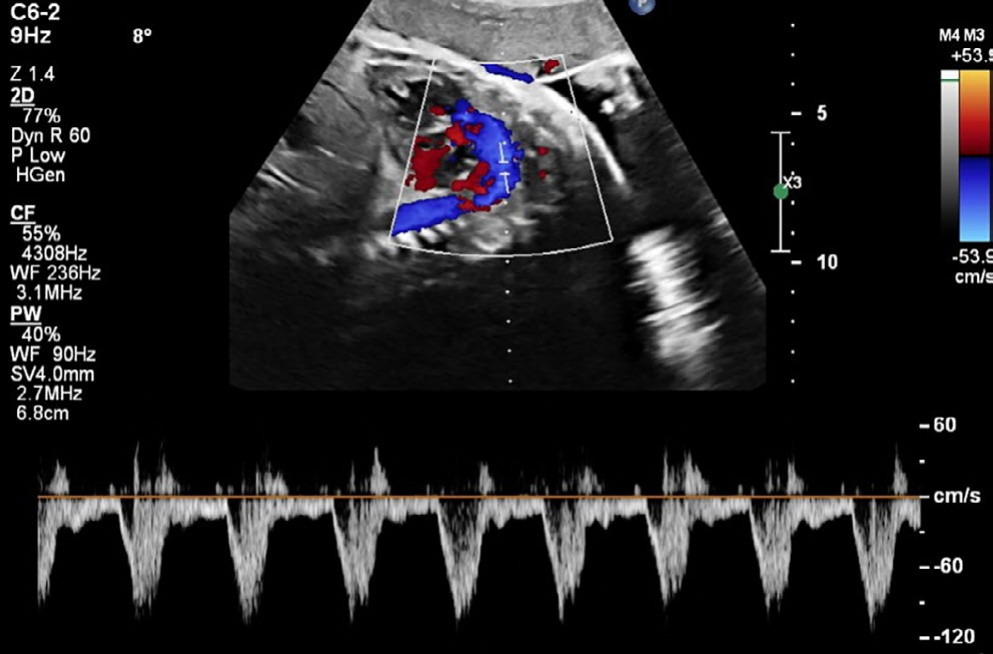
Spectral Doppler evaluation of the ductus arteriosus demonstrates decreased flow velocity, indicative of a significant reduction in stenosis severity.

A few weeks after the admission day, the axial view of the fetal cardiac ultrasound showed an obvious decline in right ventricular size and full reversion of RV‐LV discrepancy (Figure 5). Additionally, Doppler spectral placement in the stenotic portions of DA showed a significant decline in the velocity of previous stenotic portion of DA and its normalization (Figure 6).

Conclusively, this case underscores the association between sertraline and premature ductus arteriosus constriction and necessitates careful consideration and further research into safe alternatives for managing depression in pregnant patients. Enhanced awareness among healthcare providers regarding this rare complication can lead to improved outcomes for affected pregnancies. Future studies should aim to elucidate mechanisms underlying drug‐related ductus arteriosus constriction and explore safer pharmacological options for pregnant mothers.

## 5 Discussion

This case report presents a rare case of a 39‐year‐old pregnant woman who experienced premature ductus arteriosus constriction, likely linked to her chronic use of sertraline. The case is discussable from several perspectives as follows:

Perspective 1: Maternal Mental Health and Pharmacological Treatment. One critical aspect of this case is the management of the mother's mental health condition. Antidepressants like sertraline are often prescribed to manage anxiety and depression during pregnancy. While it is essential to address the mother's psychiatric needs, this case highlights the potential risks associated with certain medications. The decision to switch from sertraline to an alternative medication reflects an understanding that maternal well‐being must be balanced against fetal safety. This raises questions about how healthcare providers can better inform expectant mothers about the risks and benefits of their medications. Accordingly, a systematic review and meta‐analysis by Junkes et al. (2024) highlights an urgent need for evidence‐based guidelines regarding depression treatment during pregnancy. By systematically analyzing existing data, the authors aim to clarify which treatments are most effective and safe, ultimately guiding healthcare providers in making informed decisions [9]. Moreover, a recently published case report by Vassileva et al. (2024) also reveals a 31‐week fetus with premature ductal constriction caused by maternal treatment by sertraline in which the medical team decided to perform an emergent cesarean at 32nd gestational week [8]. Meanwhile, our study indicated that the pregnancy can be continued without a cesarian. However, the vital signs and characteristics of the fetus are also crucial to decide about an emergent caesarian.

Perspective 2: Implications for Fetal Health. The findings from fetal echocardiography indicate significant cardiovascular implications due to drug exposure in utero. The reversible nature of ductus arteriosus constriction upon discontinuation of sertraline suggests a direct relationship between maternal medication use and fetal cardiac anomalies. A study by Kolding et al. (2021) indicates that healthcare providers should weigh the benefits of treating maternal mental health conditions with sertraline against potential risks to fetal development [10]. This also emphasizes the importance of vigilant monitoring through regular ultrasounds, especially when there is known exposure to potentially harmful substances during critical periods of fetal development [11]. It also underscores the necessity for healthcare professionals to educate patients on recognizing symptoms that may warrant further investigation.

Perspective 3: Broader Context of Drug Safety During Pregnancy. This case also contributes to the broader discourse on drug safety during pregnancy. While NSAIDs are commonly cited as culprits for inducing ductus arteriosus constriction, this case introduces an SSRI into that conversation. It prompts further exploration into other classes of drugs that might pose similar risks, such as fluoxetine or beta‐adrenergic agents mentioned in the report. There is a clear need for more extensive research into how various medications impact fetal development across different gestational ages. Accordingly, emphasizes the need for caution when prescribing NSAIDs to pregnant women, particularly during the third trimester. Clinicians must weigh the benefits against potential risks such as fetal distress or other cardiovascular issues resulting from ductus arteriosus constriction [12].

Fetal echocardiography is one of the most concise methods for diagnosis of this abnormality as it verifies the presence of PDA and evaluates the shunt volume and its downstream impact [13]. However, it is essential to rule out any underlying congenital heart defects (CHDs), especially those that are duct‐dependent, prior to initiating treatment for PDA [14]. On the other hand, persistent pulmonary hypertension can be mentioned as a complication of these cases after birth; which indicates the importance of further follow‐ups [15]. Moreover, it is notable that foods that contain polyphenols (herbs, fruits, and nuts), especially if they xconsumed in the third trimester of pregnancy can have effects on the inflammatory pathway and therefore inhibition in the production of prostaglandins and finally cause ductus arteriosus constriction [16]. Also, other drugs such as fluoxetine (selective serotonin reuptake inhibitor drugs) Nephazoline (nasal spray) and Isoxsuprine (a beta‐adrenergic drug) can cause ductus arteriosus stenosis or complete obstruction [17].

## 6 Conclusion

This study highlights the link between sertraline use and premature ductus arteriosus constriction, emphasizing the need for cautious evaluation and further investigation into safer treatment options for depression during pregnancy. Greater awareness among healthcare professionals about this complication can help achieve better outcomes in affected pregnancies. Conclusively, this study raises several important considerations regarding maternal mental health, pharmacological management during pregnancy, and the potential impacts on fetal development.

## Author Contributions

**Alireza Golbabaei:** supervision, conceptualization, data gathering, writing the final draft. **Elham sadat Alavi moghaddam:** conceptualization, data gathering. **Hooman Mohammad Talebi: s**upervision, conceptualization, writing the final draft.

## Consent

A written informed consent was obtained from the patient before submission.

## Conflicts of Interest

The authors declare no conflicts of interest.

## Data Availability

Data are available via sending request to the corresponding author.
